# The emerging landscape of exosomal CircRNAs in solid cancers and hematological malignancies

**DOI:** 10.1186/s40364-022-00375-3

**Published:** 2022-05-03

**Authors:** Qinfeng Zhou, Dacheng Xie, Rong Wang, Lianfang Liu, Yue Yu, Xinyi Tang, Yongxian Hu, Dawei Cui

**Affiliations:** 1 Department of Laboratory Medicine, Zhangjiagang TCM Hospital Affiliated to Nanjing University of Chinese Medicine, Zhangjiagang, China; 2 Department of Medical Oncology, Shanghai Pulmonary Hospital & Thoracic Cancer Institute, Tongji University School of Medicine, Shanghai, China; 3 Department of Oncology, Zhangjiagang TCM Hospital Affiliated to Nanjing University of Chinese Medicine, Zhangjiagang, China; 4 Department of Health Sciences Research, Mayo Clinic, Rochester, USA; 5 Department of Laboratory Medicine, The Affiliated People’s Hospital, Jiangsu University, Zhenjiang, China; 6 Division of Hematology and Internal Medicine, Mayo Clinic, Rochester, USA; 7 Bone Marrow Transplantation Center, The First Affiliated Hospital, Zhejiang University School of Medicine, Hangzhou, China; 8 Department of Blood Transfusion, The First Affiliated Hospital, Zhejiang University School of Medicine, Hangzhou, China

**Keywords:** Circular RNA, Exosome, Exosomal circular RNA, Solid cancers, Hematological malignancies

## Abstract

Circular RNAs (circRNAs) are a type of recently discovered noncoding RNA. They exert their biological functions by competitively binding to microRNAs (miRNAs) as miRNA sponges, promoting gene transcription and participating in the regulation of selective splicing, interacting with proteins and being translated into proteins. Exosomes are derived from intracavitary vesicles (ILVs), which are formed by the inward budding of multivesicular bodies (MVBs), and exosome release plays a pivotal role in intercellular communication. Accumulating evidence indicates that circRNAs in exosomes are associated with solid tumor invasion and metastasis. Additionally, emerging studies in the last 1 ~ 2 years have revealed that exosomal circRNA also have effect on hematological malignancies. In this review, we outline the properties and biological functions of circRNAs and exosomes. In particular, we summarize in detail the mechanism and roles of exosomal circRNAs and highlight their application as novel biomarkers in malignant tumors.

## Introduction

Circular RNAs (circRNAs) are single stranded, covalently closed RNA molecules produced by the back-splicing process of pre-mRNAs, and were initially considered as splicing-related noises [1]. Nevertheless, recent researches have revealed that circRNAs may be closely related to microRNA (miRNA) inhibition, epithelial-mesenchymal transition (EMT), and tumorigenesis [2–4]. Exosomes are closely associated with circRNAs and have attracted more and more scholars’ attention in recent years. They were nanoscale membrane vesicles that were generated by most types of cells, which can convey information in the tumor microenvironment by transferring cargos such as DNA, RNA, proteins and lipids, therefore they are critical to tumor progression [5]. In the past few years, the roles of long noncoding RNAs (LncRNAs) and miRNAs derived from exosome of tumors have been extensively studied [6, 7], but exosomal circRNA has attracted increasing attention since it was first discovered in exosomes in 2015. As such, in the current review, we summarize the generation and biological functions of circRNAs and exosomes, analyze the mechanism of exosomal circRNAs in a wide variety of malignant tumors including solid cancers and hematological malignancies, and highlight their promising potential not only as diagnostic molecular markers but also as therapeutic targets.

### The properties and biological functions of circRNAs

CircRNAs were first demonstrated in RNA viruses in 1976 and subsequently discovered in the cytoplasm of eukaryotic cells and yeast [8, 9]. However, little attention has been given to exploring the value of circRNAs, as these RNAs were previously thought to be redundant products of faulty splicing [10]. With the development of newly rising bioinformatics methods and technologies, the importance of circRNAs has been gradually recognized. Subsequently, researchers have begun to determine the characteristics, biogenesis and function of circRNAs. CircRNAs, a new type of endogenous noncoding RNA (ncRNA), are described as covalent closed-loop structure without 5' caps and 3' poly (A) tails. In most cases, circRNAs are produced by back splicing of precursor messenger RNA (pre-mRNA), in which the downstream 5'-splicing donor is connected to the upstream 3'-splicing receptor by a 3'-5'-phosphodiester bond [11]. According to their composition, circRNAs are mainly classified into three categories: exon circRNAs (ecircRNAs), intron circRNAs (ciRNAs), and exon–intron circRNAs (EIciRNAs). Moreover, circRNAs have the following characteristics: (1) They are very stable and difficult to degrade with RNase R [12, 13]. (2) Their sequence is conservative to a certain extent [14]. (3) Most circRNAs are located in the cytoplasm and some are enriched in exosomes [15, 16]. (4) Most circRNAs belong to ncRNAs and are produced by exons [17]. (5) Through a selective mechanism, a single gene locus can be translated and cycled into multiple circRNAs [17].

**Fig. 1:**
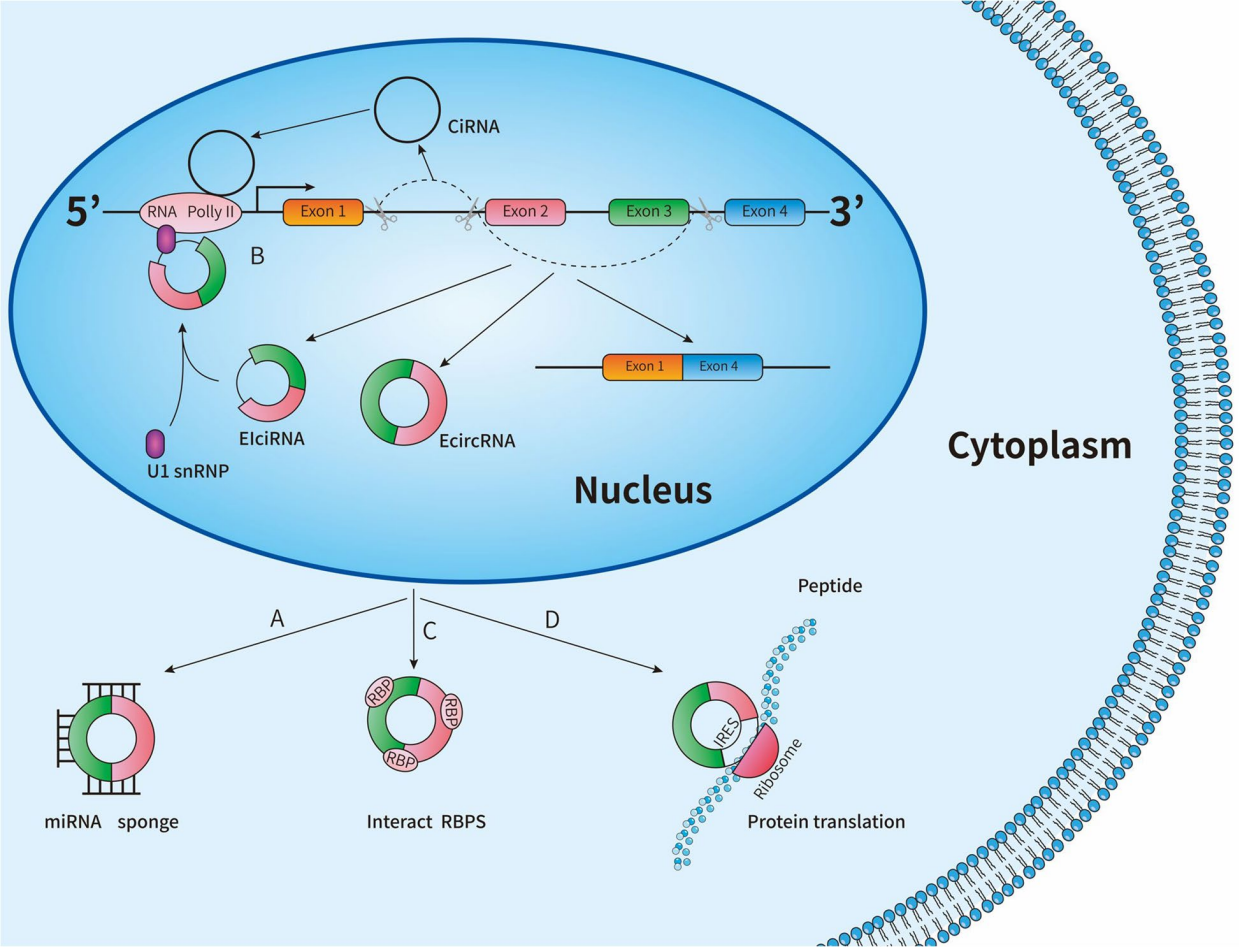
Biological functions of circular RNAs (circRNAs). **a** CircRNAs competitively bind to microRNAs (miRNAs) as miRNA sponge. **b** CircRNAs promote gene transcription and participate in the regulation of selective splicing. **c** CircRNAs interact with proteins. **d** CircRNAs translated into proteins

In addition, several potential functions of circRNAs have been reported: (1) CircRNAs competitively bind to miRNAs as endogenous competitive RNAs (ceRNAs), known as miRNA sponges (Fig. 1a). CircRNAs bind to the corresponding miRNAs via their complementary sequences, thereby regulating downstream target gene expression which is inhibited by miRNAs [18]. For example, Cirs-7 is derived from a highly conserved single exon, also known as antisense transcription of cerebellar degeneration-related protein 1 (CDR1as), which can regulate miR-7 through adsorption, thus reducing the inhibition of miR-7 on its target [19]. (2) CircRNAs promote gene transcription and participate in the regulation of selective splicing (Fig. 1b). CiRNAs are derived from introns and cannot act as sponges to absorb miRNA due to their limited number of binding sites. However, ciRNAs can enhance the expression of parental genes by regulating RNA polymerase II (RNA Pol II) [20]. EIciRNAs form the EIciRNA-U1 snRNP complex by binding to U1 small nuclear RNA (snRNA), promoting the transcription of their parent genes [21]. (3) CircRNAs can also interact with proteins (Fig. 1c). CircDnmt1 promotes nuclear translation by interacting with P53 and AUF1, leading to autophagy or reduced instability of target mRNA [22]. In addition, Memczak et al*.* confirmed that circRNA CDR1as can bind closely with AGO [18]. (4) As illustrated in Fig. 1d, some scholars also have found that circRNAs contain an open reading frame and can be translated into a polypeptide or protein [23]. CircRNAs that contain an internal ribosomal entry site (IRES) can cause ribosomal recruitment and translation initiation, while circRNAs lacking IRES cannot encode proteins [24].

### The properties and biological functions of exosome

**Fig. 2:**
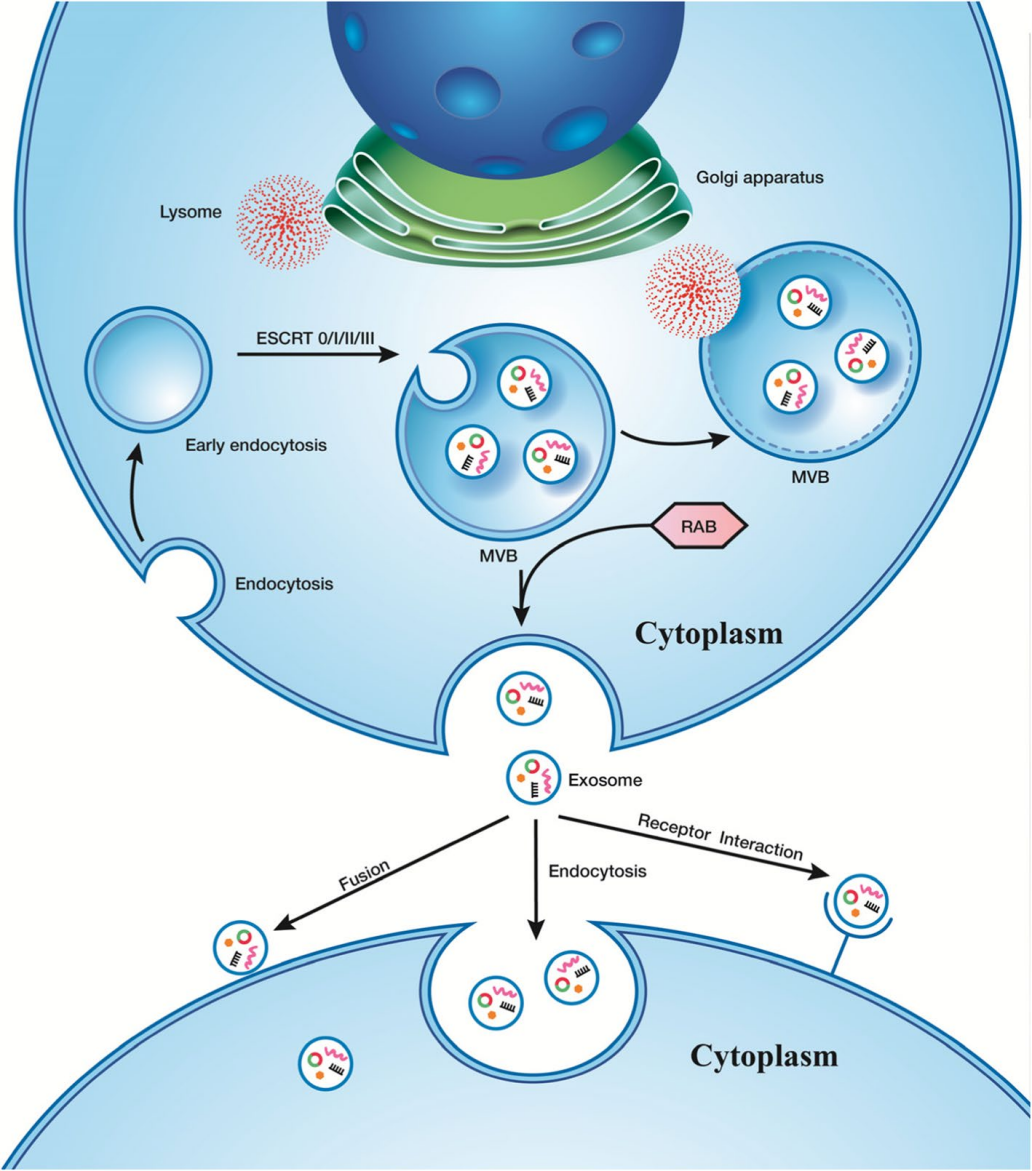
ESCRT complexes play an important role in exosome formation and secretion. Exosomes are derived from intracavitary vesicles (ILVs) that are formed by the inward budding of multivesicular bodies (MVBs), which are generated in the early to late stage of endosomal maturation. MVBs can not only fuse with lysosomes resulting in degradation of the contents, but also can fuse with plasma membranes, release exosomes into the extracellular space and facilitate intercellular communication

Exosomes were firstly discovered and characterized by Harding et al*.* and Johnstone in 1983 [25–28]. Exosomes are defined as nanoscale phospholipid double-membrane-bound vesicles that are secreted by miscellaneous types of cells into interstitial and body fluids, including, cerebrospinal fluid (CSF), urine, blood, breast milk, and saliva [29, 30]. Exosomes are 30–100 nm in diameter and have a variety of specific surface molecular markers, such as CD81, CD9, and CD63 [31]. The formation of exosomes has four stages: initiation, endocytosis, multivesicular body (MVB) formation and exosome secretion [32] (Fig. 2). Moreover, the biogenesis of exosomes involves a series of molecular mechanisms, the most important of which is the endosomal sorting complexes required for transport (ESCRT) mechanism, which is the driving factor for membrane formation and shedding [30, 33]. ESCRT complexes consist of ESCRT-0, -I, -II and -III. ESCRT-0 activates the MVB pathway and recognizes ubiquitinated proteins. ESCRT-I and ESCRT-II have contractile effects on the sprouting neck of vesicles. ESCRT-III is associated with membrane budding and vesicle shedding [34–36]. In addition, exosome secretion is regulated by different molecules; for instance, the RAB GTPase protein (e.g., RAB27A/B, RAB35 and RAB11) plays an important role in regulating intracellular vesicle transport, such as vesicle budding and movement through cytoskeletal interactions [37, 38]. Exosomes contain a variety of DNA, RNA and proteins [39]. RNAs in exosomes include mRNAs, miRNAs, LncRNAs, etc. Proteins in exosomes are divided into two categories: (1) structural proteins, such as cytoskeletal proteins, membrane transporters, Hsp70 and its molecular chaperones, signaling proteins and cytoplasmic enzymes; (2) secreted cell-specific proteins. For example, tumor-derived exosomes often contain TGF-antigens and tumor antigens [40, 41]. Exosomes from antigen-presenting cells (APCs) were detected to have some typical molecules of APCs, such as major histocompatibility complex-I (MHC-I) [42]. Studies have demonstrated that exosomes are involved in a variety of physiological and pathological processes, such as antigen presentation, angiogenesis, cell differentiation, tumor cell migration and invasion and so forth [43]. Interestingly, researchers have found that tumor cells secrete approximately 10 times as many exosomes as normal cells [44], and that cancer cells exosomes have precise targeting mechanisms, suggesting that exosomes may provide a method of cell communication and play a prominent role in tumor formation and progression [45].

### Discovery of exosomal circRNAs in solid cancers

Based on the biological characteristics of circRNAs and exosomes, more and more evidence has shown that circRNAs derived from exosomes, especially tumor cell-derived exosomes, may exert a significant biological function in different pathological and physiological processes. Li and her colleagues confirmed in 2015 that there are a huge number of stable circRNAs in exosomes [16]. The half-life time of most circRNAs are more than 48 h, while linear RNA molecules have a half-life of 20 h [12]. The reason why circRNAs have a pretty long half-life is because they are covalently closed and lack a 3' poly A tail or a 5' cap. Therefore, circRNAs are resistant to the degradation by exonuclease [4, 18]. Due to above features, circRNAs are more stable and accumulate at higher levels in cells than linear transcripts, even though linear RNAs are generated at a significantly higher rate than circRNAs [46]. In addition, Dou et al*.* evidenced that circRNAs are more abundant in exosomes than in cells and that the expression of circRNAs varies with the mutation status of KRAS (a proto-oncogene) [47]. The biological functions of exosomal circRNAs have not been fully elucidated, and an increasing number of studies have focused on exosomal circRNAs. New studies have shown that exosomes from tumor cells or other cells (such as fat cells and activated human platelets) can deliver biological information to their specific cells, achieving phenotypic changes that promote cancer invasion and metastasis [48]. For example, exosomal circ-PDE8A has been showed association with tumor progression and lymphatic invasion in pancreatic ductal adenocarcinoma (PDAC) via the miR338/MACC1/MET pathway [49]. In addition, exosomal circRNA has been observed to promote white fat browning by targeting the miR-133/PRDM16 pathway [50], which provides a new perspective for our fundamental understanding of cancer-related cachexia.

### Mechanism and roles of exosomal circRNAs in solid cancers

With an increasing number of exosomal circRNAs have been discovered and identified, there is increasing evidence that exosomal circRNAs play a key role in the development of various cancers, which including tumor proliferation, migration, invasion, etc. [51]. At present, exosomal circRNAs may regulate solid tumors through a variety of different mechanisms (Table 1): 1) Exosomal circRNAs act as miRNA sponges. 2) Exosomal circRNAs interact with proteins. 3) Possibility of exosomal circRNAs being translated into proteins. Among these mechanisms, miRNA sponge is the most common one, which regulates the expression of target genes by regulating miRNA.

**Table 1:** Mechanism and roles of exosomal circular RNA in solid cancers

Circular RNA	Expression of circRNA	Tumor types	miRNA, RBP	Downstream pathways	Biological functions	References
CircRNA_100284	up	hepatocellular carcinoma	miR-217	EZH2 and CyclinD1	Cell proliferation	[52]
Circ-DB (has_circ_0025129)	up	hepatocellular carcinoma	miR-34a	USP7/Cyclin A2	Tumor growth and metastasis	[53]
CircPTGR1	up	hepatocellular carcinoma	miR-449a	MET	Disrupt tumor microenvironmental homeostasis and promote metastasis	[54]
Circ-0000284	up	cholangiocarcinoma	miR-637	LY6E	Proliferation, migration, invasion	[55]
Circ-PDE8A	up	pancreatic ductal adenocarcinoma	miR-338	MACC1/MET/ERK or AKT pathway	Invasive growth	[49]
Circ-IARS	up	pancreatic cancer	miR-122	RhoA activity/ F-actin/ ZO-1	Vascular endothelial permeability and tumor metastasis	[56]
Circ LONP2 (hsa_circ_0008558)	up	colorectal cancer	Promote pre-miR-17 mature	Recruit DGCR8 and Drosha complex	Invasion	[57]
Circ PRMT5	up	urothelial carcinoma of the bladder	miR-30c	SNAIL1/ E-cadherin pathway	Promote pathological EMT	[58]
Circ_0044516	up	prostate cancer	miR‐29a‐3p	-	Proliferation and metastasis	[59]
CiRS-133	up	gastric tumors	miR-133	PRDM16/UCP1 pathway	Cancer cachexia	[50]
Circ-0051443	down	hepatocellular carcinoma	miR-331-3p	BAK1	Promote apoptosis and blocking cell cycle	[60]
Circ-ABCC1	up	colorectal cancer	interact with β-catenin	Wnt/β‐catenin pathway	Cell stemness, sphere formation and metastasis	[61]
Circ-CCAC1	up	cholangiocarcinoma	interact with EZH2	SH3GL2/ ZO-1/Occludin	Accelerate CCA tumorigenesis and metastasis	[62]
Has-circ_0074854	up	hepatocellular carcinoma	interact with HuR	ZEB1 signaling pathway	Activate the polarization of M2 macrophage and promote EMT of HCC cells	[63]
CircFARSA	up	non-small cell lung cancer	interact with eIF4A3	PI3K/AKT signaling pathway	Mediate polarization of M2 macrophages and promote EMT	[64]
CircLPAR1	down	colorectal cancer	interact with eIF3h	METTL3-eIF3h/ BRD4	Inhibit the proliferation, invasion and migration of CRC	[65]

Not detection

### Exosomal circRNAs act as miRNA sponges

#### CircRNA_100284

Arsenic is a toxic metal and long-term exposure can lead to tumor formation in the lungs, skin and bladder [66, 67]. Recently, Dai et al*.* found that arsenic exposure was associated with circRNA_100284 overexpression and could trigger the malignant transformation of normal hepatocytes and accelerate cell cycle and proliferation [52]. MiR-217 is a tumor suppressor involved in a variety of cancers, including liver cancer [68]. CircRNA-100284 can act as a miRNA sponge of miR-217 and stimulate the downstream signaling pathway, leading to an increase in Enhancer of Zeste Homolog 2 (EZH2) and CyclinD1 enhancer expression, leading to the abnormal proliferation of liver cells. Furthermore, Dai et al*.* demonstrated with electron microscopy that exosomes carrying circRNA_100284 were transferred from malignant cells to nonmalignant cells, leading to the malignant transformation of normal cells [52].

#### Circ-DB

Circ-DB (circ-deubiquitination, has_circ_0025129) is an adipose secreted exosomal circRNA that is upregulated in hepatocellular carcinoma (HCC) patients with high body fat ratio [53]. Ubiquitin-specific protease 7 (USP7) is a deubiquitinating enzyme that plays a key role in the cell cycle, proliferation and DNA repair [69, 70]. High levels of USP7 are often found in HCC tissues and are associated with tumor growth and invasion, leading to poor overall survival [71, 72]. Studies have shown that exosomal circ-DB secreted by adipose cells can inhibit miR-34a by targeting miR-34a and activate deubiquitination-related USP7, reducing DNA damage and promoting the proliferation and migration of HCC cells by activating the USP7/Cyclin A2 signaling pathway, leading to tumor growth and metastasis in vivo [53].

#### CircPTGR1

A study showed that the increased metastatic ability of HCC cells could be gifted to cells with low or no metastasis potential by exosomes carrying circPTGR1, thus promoting the metastasis ability and progression of HCC cells [54]. CircPTGR1 is abundant in exosomes from serum of HCC patients and is related to clinical stage and prognosis. CircPTGR1 from exosomes with high metastatic potential and abundance in cells may inhibit the interaction between miR-449a and MET in recipient cells, thus having effects on cells with low metastatic potential, disrupting tumor microenvironment homeostasis, and triggering HCC progression [54].

#### Circ-0000284

Circ-0000284 is an isoform of circHIPK3 and is involved in tumor progression [55]. Wang et al*.* showed that circ-0000284 could absorb miR-637 and promote the proliferation, migration, and invasion of cholangiocarcinoma (CCA) cells. Circ-0000284 has the ability to sponge miR-637, which contributes to increased expression of lymphocyte antigen-6E (LY6E) through competitive binding of miR-637. Interestingly, the LY6 family has been reported to have increased expression in many cancers including colorectal cancer (CRC), glioma, ovarian cancer, gastric cancer, breast cancer and lung cancers. Moreover, high LY6E expression has been found to be significantly related to poor clinical outcomes and poorer overall and disease-free survival than low LY6E expression [73]. In addition, miR-637 can also influence CCA progression by targeting transcripts of other coding proteins involved in cell proliferation and migration, such as CDK6, STAT3, and AKT1 [74].

#### Circ-PDE8A

PDAC is the most serious malignancy in humans and its metastatic potential and risk of recurrence, resulting in a very high mortality rate [75]. In pancreatic cancer, the tumor-released exosomal circRNA PDE8A promotes aggressive growth through the miR-338/MACC1/MET pathway [49]. In this study, Li et al*.* detected the exosomal circRNA expression profile of PDAC cells with liver metastasis by microarray technology and found that high circ-PDE8A expression was associated with lymphoid infiltration, tumor-node-metastasis (TNM) stage, and poor survival in PDAC patients. Further studies showed that circ-PDE8A promoted the invasive growth of PDAC cells by upregulating MET. Circ-PDE8A, as the ceRNA of miR-338, regulates MACC1 and stimulates invasive growth through the MACC1/MET/ERK or AKT pathways. Finally, the expression of circ-PDE8A in plasma exosomes of PDAC patients was analyzed and implicated in the progression and prognosis of PDAC patients [49]. Therefore, circ-PDE8A may play a pivotal role in tumor invasion.

#### Circ-IARS

**Fig. 3:**
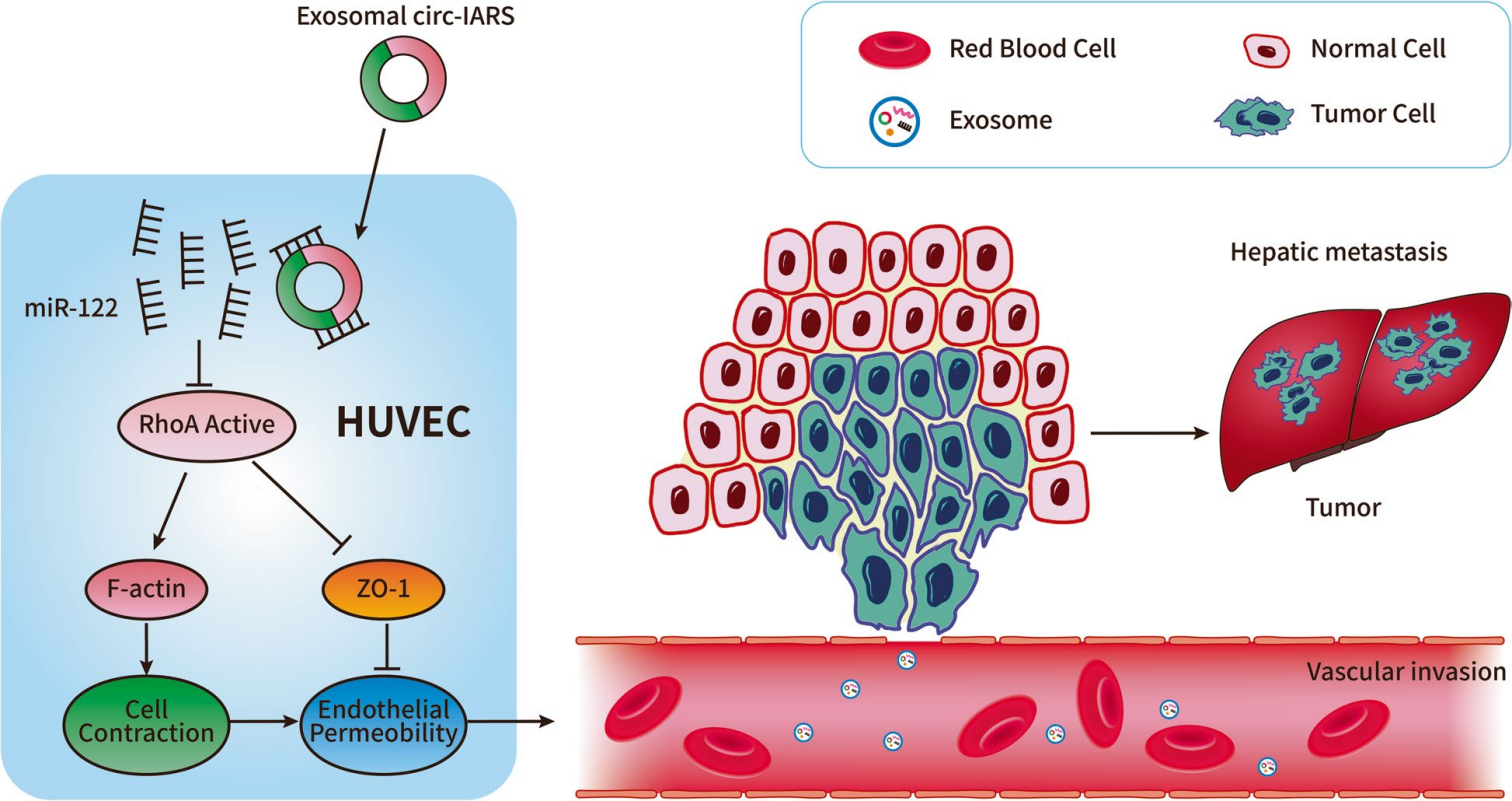
The role of circ-IARS in tumor invasion and metastasis. Overexpression of circ-IARS can competitively adsorb miR-122 that alleviates its inhibition of RhoA activity, which increases F-actin expression and promotes cell contraction. Moreover, increasing RhoA activity reduced the expression of ZO-1, which disrupted the tight connection with endothelial cells, and eventually led to increased vascular endothelial permeability and promoted tumor metastasis

Circ-IARS is a novel circRNA occurred through the progression of pancreatic cancer [56]. Li and his colleagues showed that circ-IARS entered human microvascular endothelial cells (HUVECs) through exosomes and promoted tumor invasion and metastasis [56] (Fig. 3). Liver metastasis, vascular infiltration and lymph node metastasis stage were sped up by the increased expression of circ-IARS. As a result, the postoperative survival time decreased. Previous studies have reported increased activity of RhoA, a member of the Ras homologous gene family in HUVECs, which can promote actin cytoskeleton remodeling and cell contraction and can reduce the expression of tight junction ligand protein Zonula occludens-1 (ZO-1) [76, 77], resulting in endothelial barrier dysfunction [78–80]. Studies have found that overexpression of circ-IARS can competitively adsorb miR-122 that alleviate its inhibition of RhoA activity of downstream target gene, which increases F-actin expression and promotes cell contraction [56]. Moreover, increasing RhoA activity reduced the expression of ZO-1, which disrupted the tight connection with endothelial cells, and eventually led to increased vascular endothelial permeability and promoted tumor metastasis [56].

#### CircLONP2

CircLONP2 (hsa_circ_0008558) is critical for the metastasis of CRC cells and is closely related to the aggressive clinicopathological features of CRC patients [57]. In addition, circLONP2 directly acts on and facilitates primary miRNA-17 (pre-miR-17) processing by recruiting DiGeorge syndrome critical region gene 8 (DGCR8) and the Drosha complex in a DDX1-dependent manner. Moreover, when mature miR-17-5p is upregulated, it can be assembled into exosomes and transferred to adjacent cells, leading to the proliferation of metastasis initiation among primary CRC cells, thus enhancing their invasion ability. The study further confirmed that CRC exosomes containing miR-17-5p mimics conferred higher migration and invasion abilities, while exosomes of miR-17-5p inhibitors conferred the opposite effect. These results suggest that upregulated circLONP2 is an important metastasis driver of CRC and may serve as a new prognostic biomarker and potential anti-metastatic therapeutic target in clinical practice.

#### CircPRMT5

Chen et al*.* reported that circPRMT5 is overexpressed in both the serum and urine of patients with urothelial carcinoma of the bladder (UCB) and is positively correlated with lymph node metastasis and tumor progression [58]. Further studies have shown that circPRMT5 can act as miR-30c sponge to promote the pathological EMT of UCB cells, thereby enhancing the level of the target genes SNAIL1 and E-cadherin, making cells become more aggressive via the circPRMT5/miR30c/SNAIL1/E-cadherin pathway [58, 81]. This suggests that circPRMT5 may be a promising prognostic biomarker for UCB patients.

#### Circ_0044516

Circ_0044516 is extremely upregulated in the exosomes and cells of prostate cancer patients [59]. Circ_0044516 acts as a sponge of miR‐29a‐3p. Bioinformatics and luciferase reporter assays confirmed that circ_0044516 down-regulated the expression of miR-29a‐3p, which was negatively correlated with miR-29a‐3p expression in prostate cancer. Colony formation experiments showed that the downregulation of circ_0044516 resulted in a decrease in the number of prostate cancer cell colonies. In addition, data showed that the expression of circ_0044516 in the blood exosomes of patients with highly metastatic prostate cancer was higher than that in those of patients with prostate cancer of low metastatic potential, and circ_0044516 also decreased the migration ability of prostate cancer cells [59]. The above studies show that circ_0044516 suppress the proliferation and metastasis of prostate cancer cells.

#### CiRS-133

Cancer-related cachexia is a metabolic syndrome and is a negative cancer patient survival risk factor [82]. Cachexia is typically characterized by excessive energy expenditure compared to that in healthy individuals [83, 84]. However, the molecular mechanisms of cancer-related cachexia are poorly understood. Zhang et al*.* found that exosomal circRNAs from gastric tumors promoted white fat browning by targeting the miR-133/PRDM16 pathway [50]. They identified a cachexia-related plasma exosome ciRS-133 and found that exosomes derived from GC cells delivered ciRS-133 to preadipocytes, activating PRDM16/UCP1 by inhibiting the biological function of miR-133 and promoting the differentiation of preadipocytes into brown-like cells. In addition, knockdown of ciRS-133 reduced cancer cachexia and oxygen consumption and heat production in tumor-implanted mice. Thus, exosome-delivered ciRS-133 is an involved factor in white adipose browning and plays a key role in cancer-related cachexia.

#### Circ-0051443

Normal cells could transmit exosomal circ-0051443 to HCC cells to inhibit malignant biological behaviors by inducing apoptosis and blocking cell cycle progression [60]. BAK1 is an important regulator of cell death that interacts with proteins to initiate mitochondrion-mediated apoptosis. Previous studies have found that BAK1 is correlated with the development of a variety of tumors, including pediatric germ cell tumors [85], cervical cancer [86], chronic lymphocytic leukemia [87], and non-small cell lung cancer (NSCLC) [88]. Studies have shown that BAK1 expression is regulated by exosommal circ-0051443 through competitive binding of miR-331-3p, which thereby inhibit the malignant biological behavior of HCC [60]. Exosomal circ-0051443 can be used as a predictor and potential therapeutic target for HCC.

### Exosomal circRNAs interact with proteins

#### Circ‐ABCC1

Studies have shown that exosomes from CD133^+^ CRC cells significantly enhance cell stemness, sphere formation and metastasis [61].What’s more, circ‐ABCC1 overexpression was contributed to tumorigenesis of CD133^−^/HCT15 or CD133^−^/Caco2 cells. Researchers also confirmed that circ-ABCC1 could interact with β-catenin into the cell nucleus and activate the Wnt/β-catenin pathway to regulate CRC progression by RIP and RNA pull down assays [61]. Overall, cell stemness and metastasis would be mediated by exosomes from CD133^+^ cells carrying circ‐ABCC1 in CRC, indicating that circ‐ABCC1 may act as a new candidate target for CRC treatment.

#### Circ-CCAC1

Xu et al*.* found that the level of exosomal circ-CCAC1 in serum of patients with CCA was higher than that of patients with benign hepatobiliary disease [62]. Exosomal circ-CCAC1 of CCA cell can be transferred to endothelial cells, damaging the endothelial barrier and inducing angiogenesis. RIP and RNA pull-down assays showed that there was an obvious interaction between circ-CCAC1 and EZH2, and the high expression of circ-CCAC1 significantly increased the cytoplasmic localization of EZH2, inhibiting the binding of EZH2 to the SH3GL2 promoter and H3K27 trimethylation level, thereby resulting in the increased expression of SH3GL2. Moreover, SH3GL2 reduced the level of intercellular junction proteins by negatively regulating ZO-1/Occludin. In addition, exosomal circ-CCAC1 overexpression accelerates CCA tumorigenesis and metastasis in vivo [62]. Therefore, blocking exosome circ-CCAC1 transmission may be a future treatment strategy for CCA, which needs to be further explored.

#### Has-circ_0074854

HuR is an RNA binding protein (RBP), is known to stabilize mRNAs by preventing gene degradation. Wang et al*.* found that has-circ_00074854 interacts with HuR through RNA pull-down analysis [63]. Down regulation of has_circ_00074854 further inhibited the migration, invasion and EMT of HCC cells by reducing the stability of HuR protein and inhibiting ZEB1 signaling pathway. In addition, they also found that HCC cell-derived exosomes can induce activation of M2 macrophages and promote migration, invasion and EMT of HCC cells. And the down-regulated exosome of hsa_circ_00074854 inhibited the polarization of M2 macrophage, thus inhibiting the migration, invasion and EMT of HCC cells in vitro and in vivo [63]. In summary, this study provided the evidence that exosomal hsa_circ_00074854 can excert a biological role by interacting with HuR in HCC.

#### CircFARSA

Another study also reported that exosomal circRNA can promote tumor metastasis and EMT by mediating polarization of M2 macrophages. Chen et al*.* found that circFARSA was significantly upregulated in NSCLC tissues, and the NSCLC cell-derived exosomal circFARSA could polarize the macrophages to a M2 phenotype [64]. In vitro experiments, macrophages pretreated by exosomal circFARSA were co-cultured with NSCLC cells, which could promote NSCLC cell metastasis and EMT. They explored the mechanism by which exosomal circFARSA induced polarization of M2 macrophages and found that circFARSA activated the PI3K/AKT signaling pathway through PTEN ubiquitination and degradation. More interestingly, they found that an RBP named eIF4A3 can bind to the upstream and downstream sequences of circFARSA mRNA in NSCLC cells, which could promote circFARSA synthesis. However, they did not verify the effect of exosomal circFARSA on NSCLC tumor size in vivo, and further exploration is needed in the future.

#### CircLPAR1

The latest study, published in 2022, showed that exosomal circLPAR1 was significantly down-regulated in the plasma of CRC patients and could be successfully detected [65]. ROC curve showed that exosomal circLPAR1 could be used to distinguish CRC patients from non-cancer controls, and the combination of exosomal circLPAR1 with CRC diagnostic markers CEA and CA19-9 could improve the AUC value. Furthermore, exosomal circLPAR1 acts as a sponge for eIF3h to influence BRD4 translation. It can directly bind to eIF3h to inhibit METTL3-eIF3h interaction and further down-regulate the level of BRD4, thus inhibiting the proliferation, invasion and migration of CRC cells. Meanwhile, circLPAR1 can inhibit the growth of CRC in vivo experiments [65]. In this study, the specific mechanism of exosomal circLPAR1 in CRC was demonstrated. The convenience and stability of the detection of exosomal circLPAR1 in the plasma of patients opens up a new way for the diagnosis of CRC.

### Possibility of exosomal circRNAs being translated into proteins

In recent years, IRES and N6-methyladenosines (m6A)-mediated cap-independent translation initiation have been identified as potential mechanisms for circRNA translation [89]. To date, several translated circRNAs have been suggested that they could play key roles in cancers. CircSHPRH can encode a new 146 amino acids protein (SHPRH-146aa) in glioma, which shows tumor suppressive activity during tumorigenesis [90]. CircFBXW7 has also been reported to inhibit the proliferation and migration of triple-negative breast cancer (TNBC) cells by encoding a 185 amino acids peptide (FBXW7-185aa) as a tumor suppressor [91]. CircLINC-PINT inhibits the proliferation of glioblastoma cells by encoding a peptide containing 87 amino acids (PINT87aa) [92]. CircPPP1R12A encodes a 73 amino acids peptide (CircPPP1R12A-73aa) to activate the Hippo-YAP signaling pathway, which promotes the proliferation and metastasis of colon cancer [93].

Although these reported proteins of circRNAs translation play important roles in cancers, the research on circRNA translation is still in its infancy. The various circRNAs of translation in a variety of other tumors need to be further explored. In addition, we found that the mechanism of circRNAs in exosomes derived from tumors have been mostly acting as microRNA sponges since 2015, then followed by interacting with proteins in the last 1 ~ 2 years. However, the regulation of tumor development by circRNAs translating into proteins in exosomes has not been reported. Whether the exosomal circRNAs can take advantage of exosomal targeting merit to deliver their encoding proteins into recipient cells or translate into proteins after circRNAs entering recipient cells, thus playing a role in tumor progression needs further exploration and confirmation. We believe that the translation of exosomal circRNAs will provide a new perspective and approach for the diagnosis and treatment of cancer.

### Exosomal circRNA also implicate in hematological malignancies

In previous studies, the vast majority of exosomal circRNA were associated with solid tumors. Emerging studies have revealed that the expression of circRNAs is also strongly associated with tumorigenesis and prognosis of hematological malignancies. Exosome can be used as a signal molecule in bone marrow microenvironment and is closely related to the occurrence and development of hematological malignancies. However, the effect of exosomal circRNA on hematological malignancies has only been found and reported in the last 1 ~ 2 years. Therefore, we have also summed up exosomal circRNA implicated in hematological malignancies. It was found that the reported exosomal circRNAs in hematological malignancies were mainly concentrated in several diseases such as Acute myeloid leukemia (AML) [94, 95], Chronic lymphocytic leukemia (CLL) [96] and Multiple myeloma (MM) [97, 98] (Table 2).

**Table 2:** Exosomal circRNA implicate in hematological malignancies

Diseases	Exosomal circRNA	Levels	miRNAs, RBPs, and pathways targeted	Impact on Hematologic disease	References
AML	circ_0004136	up	miR-570-3p/TSPAN3	Increase AML cell viability, cell cycle progression, migration and invasion and decreased apoptosis	[94]
AML	circ_0009910	up	miR‐5195‐3p/GRB10	Increase proliferation, apoptosis and cell cycle progression of AML cells	[95]
CLL	mc-COX2	up	-	Dampen mitochondrial functions and regulates CLL cell growth	[96]
MM	circMYC	up	-	Associate with the recurrence and Bortezomib resistance	[97]
MM	chr2:2,744,228–2,744,407 +	up	hsa-miR-6829-3p/GRIN2B	Neuronal cell death and MM-related PN	[98]
MM	circ-G042080	up	hsa-miR-4268/TLR4	Associate with MM-related myocardial damage and increase autophagy level in cardiomyocytes	[99]

Not detection

### Exosomal circRNA in AML

AML is accounting for approximately 20% of pediatric leukemia [100, 101]. Bi et al*.* first reported one exosomal circRNA (circ_0004136) in AML progression [94]. Functional analyses such as dual-luciferase reporter, RNA immunoprecipitation assays, Cell Counting Kit-8, transwell and flow cytometry assay suggested that exosomal circ_0004136 knockdown hampered AML cell viability, cell cycle progression, migration and invasion, and promoted cell apoptosis by targeting the miR570-3p/TSPAN3 axis, highlighting a novel therapeutic strategy for AML management [94].

Hsa_circ_0009910 (circ_0009910) is a novel leukemia‐related circular RNA [102, 103], and expression of circ_0009910 is upregulated in AML bone marrows, cells, and exosomes [95]. MiR‐5195‐3p acted as the sponge of circ_0009910 and growth factor receptor‐bound protein 10 (GRB10) acted as downstream targets of miR‐5195‐3p were confirmed by dual‐luciferase reporter assay and RNA immunoprecipitation [95]. Circ_0009910 could be shuttled via exosomes to mediate proliferation, cell cycle and apoptosis of AML cells by regulating miR‐5195‐3p/GRB10 axis.

### Exosomal circRNA in CLL

CLL is the most popular incurable B cell neoplasm [104]. However, the functions and clinical significance of circRNAs in CLL have been rarely studied. Wu et al*.* demonstrated for the first time that a novel circRNA, mc-COX2, generated from the COX2 gene on the mitochondrial genome, was highly expressed in the plasma exosomes of CLL patients and might be closely related to prognosis [96]. In addition, mc-COX2 impacts mitochondrial functions and regulate CLL cell proliferation and apoptosis. On the contrary, carbonyl cyanide 3-chlorophenylhydrazone (CCCP), one inhibitor of mitochondria functions, can dramatically downregulated the levers of mc-COX2 [96]. However, the mechanisms of mc-COX2 regulating mitochondrial and affecting the progress of the disease have not reported, which needs to be further explored.

### Exosomal circRNA in MM

MM is the second most popular hematological malignancy and the most common type of human plasma cell disease [105]. Major clinical symptoms of MM are anemia, renal failure, bone destruction, hypercalcemia and cardiac comorbidities [106].

Although some drug especially bortezomib (BTZ) has been a successful chemotherapeutic for MM, some patients still have failed in treatment due to BTZ drug resistance [107]. Therefore, the search for new markers has important clinical significance for the development of new treatments for MM. Luo et al*.* found that the expression of CircMYC was increased in serum exosomes of MM patients [97]. Moreover, patients with BTZ resistance were found to have higher exosomal circMYC expression than that before resistance. Furthermore, high exosomal circMYC levels were independent predictors of poor prognosis in patients with MM by using univariate and multivariate Cox regression analysis and had lower overall survival and progression-free survival, which implicated that exosomal circMYC have potential clinical application as a biomarker for the diagnosis and prognosis of MM [97].

Peripheral neuropathy (PN) is an incurable complication of MM [108]. Zhang et al*.* firstly identified that a novel exosomal circRNA, chr2:2,744,228–2,744,407 + , could have potential to be an excellent therapeutic target for MM-related PN [98]. They found that chr2:2,744,228–2,744,407 + were highly expressed in the exosome of serum in patient with MM-related PN, and applied bioinformatics analysis to predict that chr2:2,744,228–2,744,407 + might induce MM-related PN via the downstream hsa-miR-6829-3p/GRIN2B axis [98]. What’s more, ROC curve, univariate and multivariate COX regression analysis showed that this exosomal circRNA could be an independent prognostic factor in MM-related PN [98]. However, the mechanism of this exosomal circRNA in MM needs to further verification and exploration.

Myocardial damage is another mostly incurable complication of MM. Sun et al*.* also demonstrated that 1265 upregulated circRNAs and 787 downregulated circRNAs have significant differences, which were identified in serum exosomes of MM patients compared with healthy controls by high-throughput sequencing [99]. They found that the upregulated circ-G042080 was closely associated with MM-related myocardial damage by using bioinformatics analysis including GO and KEGG analyses. In addition, they also predicted and verified the downstream miRNAs and target genes of circ-G042080, demonstrated that there was a circ-G042080/hsa-miR-4268/TLR4 ceRNA axis in H9C2 cells incubated with exosomes from MM and can induce autophagic death in cardiomyocytes [99]. More importantly, circ-G042080 was found to be positively correlated with TnT and proBNP levels which represented the clinical features of heart damage. Meanwhile, the ROC curve and Cox analyses suggested that circ-G042080 could be used as an effective clinical prognostic indicator and independent prognostic factor of patients with MM-related myocardial damage [99].

### Application of exosomal circRNAs as biomarkers in malignant tumors

Noninvasive early detection of malignant tumors has always been a hot topic in tumor research. It is very important to find sensitive and specific biomarkers for the early diagnosis and treatment of tumors. Some studies have shown that microRNAs and long noncoding RNAs can act as biomarkers for tumors [109, 110]. CircRNAs are abundant, conserved, and stable, and are insensitive to RNase and these features highlight the possibility that circRNA could become predominant markers for disease. According to current publications, the potential of circRNAs as recognized biomarkers in clinically relevant diseases is being widely explored. For example, the presence of circRNA in exosomes (exo-hsa_circRNA) was found to be associated with CXCR4 expression which was confirmed to be related to the lymph node metastasis of lung adenocarcinoma in vivo, so it may be a predictor of lymph node metastasis in lung adenocarcinoma [111]. PDAC survival curve analysis showed that high expression of circRNA PDE8A in exosomes was associated with low survival rates. Exosomal circ-PDE8A may be a useful marker for PDAC diagnosis or prognosis prediction [49]. Circ 0,044,516 in exosomes promotes the proliferation and metastasis of prostate cancer cells, which suggests that exosomal circ 0,044,516 is a potential biomarker [59]. Hsa-circ-0004771 in exosomes was significantly upregulated in the serum of CRC patients compared to normal controls and had a significant diagnostic effect, which may provide new opportunities for potential CRC diagnosis strategies [112].

Recent studies have shown that exosomal circRNAs also mediate chemotherapy-resistance or radio resistance in various cancers. Some researchers observed circ-Cdr1as was downregulated in serum exosomes of cisplatin-resistant ovarian cancer patients [113]. Zhang et al.demonstrated exosomal Circ-XIAP can promote Docetaxel (DTX) resistance in prostate cancer by regulating miR-1182/TPD52 axis. Exosomal circ-XIAP is overexpressed in DTX-resistant cells, and down-regulation of circ-XIAP can enhance the sensitivity of DTX [114]. Ding et al. found that exosomal CircNFIX was up-regulated in serum of patients with temozolomide (TMZ) chemoresistance of glioma, which could promote the growth of glioma, and its depletion enhanced the sensitivity of glioma cells to TMZ in vivo [115]. Zhao et al*.* analyzed circRNAs in the extracellular vesicles (EVs) of U251 and radioresistant U251 cells by the RNA Seq method and found that exosomal circRNAs have an effect on radio resistance [116]. CircATP8B4 was proven to facilitate glioma radio resistance by serving as a miR-766 sponge, suggesting that circATP8B4 in EVs may be emerged as a potential biomarker for glioma radio resistance. Therefore, exosomes carrying circRNAs and engineered siRNAs targeting specific circRNAs may be valuable not only in accurate and effective treatment but also in drug development to suppress tumor progression [117]. These siRNA molecules can downregulate the expression of circRNA and indirectly inhibit circRNA-induced injury. For example, inhibition of circRNA-ACAP2 and circCCDC66 by siRNA transfection of colon cancer cells resulted in decreased cell proliferation, migration, and invasion rates compared to those in the control and NC groups [118, 119]. In addition, sensitive cells could receive ciRS-122 through exosomes from oxaliplatin resistant cells, then their glycosylation and drug resistance would be promoted through miR-122 sponging and PKM2 upregulation. Moreover, si-ciRS-122 transported by exosomes could inhibit glycolysis and reverse oxaliplatin resistance by regulating the ciRS-122/miR-122/PKM2 pathways in vivo, thus enhancing the response to antitumor drugs [120].

## Conclusions

CircRNAs are endogenous, abundant, conserved, and stable regulatory RNAs, which can play biological functions through a variety of mechanisms. Exosomes carry specific proteins and RNA cargos and have identified as a new method for intercellular communication [121, 122]. In recent years, exosomal circRNAs have become a new field and hotspot in malignant tumors research. As such, in this review, we mainly focused on the multiple mechanisms of exosomal cicRNAs playing important roles in tumors: miRNA sponging, circRNA-protein interactions, and translation into proteins. We also elaborated the role and application of exosomal circRNAs in tumor development, including regulation of cell proliferation, invasion, migration, metastasis, and drug resistance. Due to high specificity and stability of exosomal circRNAs, they may become potential malignant tumors biomarkers for early detection and may accurately predict the most appropriate treatments for patients. Furthermore, they may have the capability to monitor disease progression or recurrence. Unlike biomarkers found in tumor tissue, exosomal circRNAs can be easily detected in peripheral blood and are much easier to obtain from patients. Intriguingly, the influence of exosomal circRNA on hematological malignancies, which is a very novel area and not confined to solid tumors, has also been gradually concerned, including hematological malignant tumor cell viability, cell cycle progression, migration and invasion, apoptosis, recurrence and drug resistance. Therefore, we believe that exosomal circRNA may be more promising as clinical diagnostic markers.

It is important to note that exosomes are natural drug carriers for targeted therapy of malignant tumors. Although many studies have assessed the role of exosomal circRNAs in tumors, relatively few studies have assessed the specific function of exosomal circRNAs. Moreover, recent studies have shown that tumor-derived exosomes can regulate dendritic cell differentiation and maturation or macrophage polarization by delivering their cargos to surrounding immune cells in the tumor microenvironment, thus affecting tumor growth. The specific mechanism and in vivo experimental studies need to be further explored and improved. In addition, interestingly, cells also can clear circRNAs via exosomes. Further studies are yet to elucidate the detailed mechanism of exosomal circRNA clearance and release. With the continuous exploration of these unknown factors, we believe that relevant studies on exosomal circRNAs will reveal new opportunities for clinical diagnosis strategies and provide new methods for malignant tumors treatment.

## Data Availability

Not applicable.

## Abbreviations

circRNAs: Circular RNAs
miRNAs: MicroRNAs
ILVs: Intracavitary vesicles
MVBs: Multivesicular bodies
EMT: Epithelial-mesenchymal transition
LncRNAs: Long noncoding RNAs
ncRNA: Noncoding RNA
ecircRNAs: Exon circRNAs
ciRNAs: Intron circRNAs
EIciRNAs: Exon–intron circRNAs
ceRNAs: Competitive RNAs
CDR1as: Cerebellar degeneration-related protein 1
RNA Pol II: RNA polymerase II
snRNA: Small nuclear RNA
IRES: Internal ribosomal entry site
CSF: Cerebrospinal fluid
ESCRT: Endosomal sorting complexes required for transport
ALIX: ALG-2 interaction protein X
APCs: Antigen-presenting cells
MHC-I: Major histocompatibility complex-I
PDAC: Pancreatic ductal adenocarcinoma
EZH2: Enhancer of Zeste Homolog 2
HCC: Hepatocellular carcinoma
USP7: Ubiquitin-specific protease 7
CCA: Cholangiocarcinoma
LY6E: Lymphocyte antigen-6E
CRC: Colorectal cancer
TNM: Tumor-node-metastasis
HUVECs: Human microvascular endothelial cells
ZO-1: Zonula occludens-1
DGCR8: DiGeorge syndrome critical region gene 8
UCB: Urothelial carcinoma of the bladder
NSCLC: Non-small cell lung cancer
RBP: RNA binding protein
TNBC: Triple-negative breast cancer
AML: Acute myeloid leukemia
CLL: Chronic lymphocytic leukemia
MM: Multiple myeloma
GRB10: Growth factor receptor‐bound protein 10
CCCP: Carbonyl cyanide 3-chlorophenylhydrazone
BTZ: Bortezomib
PN: Peripheral neuropathy
DTX: Docetaxel
TMZ: Temozolomide
EVs: Extracellular vesicles
